# Analogies between Vanadoborates and Planar Aromatic Hydrocarbons: A High-Spin Analogue of Aromaticity

**DOI:** 10.3390/ma11010015

**Published:** 2017-12-23

**Authors:** R. Bruce King

**Affiliations:** Department of Chemistry, University of Georgia, Athens, GA 30602, USA; rbking@chem.uga.edu

**Keywords:** vanadium, boron, vanadoborates, planar aromatic hydrocarbons, aromaticity

## Abstract

The vanadium-vanadium interactions in the polygonal aggregates of d^1^ vanadium(IV) atoms, with a total of 4*k* + 2 vanadium electrons (*k* an integer) imbedded in an electronically inactive borate matrix in certain vanadoborate structures are analogous to the ring carbon-carbon interactions in diamagnetic planar cyclic hydrocarbons. They thus represent a high-spin analogue of aromaticity. Thus, the vanadoborate anion [V_6_B_20_O_50_H_8_]^8−^ with six V(IV) electrons (i.e., 4*k* + 2 for *k* = 1) contains a macrohexagon of d^1^ V(IV) atoms with four unpaired electrons. This high-spin system is related to the low-spin aromaticity in the diamagnetic benzene having six π electrons. Similarly, the vanadoborate anion [V_10_B_28_O_74_H_8_]^16−^ with ten V(IV) electrons (i.e., 4*k* + 2 for *k* = 2) contains a macrodecagon of d^1^ V(IV) atoms with eight unpaired electrons. Again, this high-spin system is related to the aromaticity in the diamagnetic 1,6-methanol[10]annulene, having ten π electrons.

## 1. Introduction

In 2001, I surveyed aromaticity in transition metal oxide structures in a publication based on a presentation at a mathematical chemistry conference [1]. At that time, some particularly interesting chemistry of vanadoborates was beginning to emerge. A key species is the ion [V_6_B_20_O_50_H_8_]^8−^ shown by X-ray crystallography to have a macrohexagon of d^1^ vanadium(IV) ions as V–O–V units imbedded into an electronically inactive borate matrix [2,3]. In this V(IV) macrohexagon, the interaction between the single d electrons of each vanadium atom is mathematically similar to the interaction between the single π electrons provided by each CH vertex in benzene. However, because of the oxygen spacers between the vanadium atoms in [V_6_B_20_O_50_H_8_]^8−^, these vanadium-vanadium interactions are too weak to completely overcome electron-electron repulsion. This prevents complete spin pairing in [V_6_B_20_O_50_H_8_]^8−^, thereby leading to a paramagnetic species in contrast to the diamagnetic benzene. The implied the possibility of interactions between transition metals separated by oxygen bridges in polyoxometalate structures relates to the 1988 experimental observation by Baker and co-workers of ring currents in wholly inorganic heteropolyoxometalate blue structures using a modification of Evans’ susceptibility method [4].

The field of vanadoborates has expanded significantly since the publication of my 2001 article in view of their potential applications as building blocks for the construction of mesoporous frameworks, including materials with unusual magnetic properties. Typically, hydrothermal reactions have been used for their synthesis. Of particular interest as a new high-spin aromatic system is the [V_10_B_28_O_74_H_8_]^16−^ anion, in which a V_10_ macrodecagon is imbedded into a borate matrix [5,6,7,8], making it a high-spin analogue of the the hydrocarbon cyclodecapentaene, also known as [10]annulene [9]. In this paper, I review the vanadium-vanadium interactions in the V_6_ macrohexagon in [V_6_B_20_O_50_H_8_]^8−^ mentioned briefly in my earlier article [1]. I then show how related concepts suggest an analogue of aromaticity in the V_10_ unit of [V_10_B_28_O_74_H_8_]^16−^, thereby making it a high-spin analogue of the experimentally known hydrocarbon [10]annulene (cyclodecapentaene).

## 2. Aromaticity in Polyoxometalates

The concept of aromaticity was initially applied to a specific class of organic substances approximately two centuries ago, when chemical bonding in general was very poorly understood [10]. The initial aromatic substances, notably benzene, first obtained by pyrolysis of whale oil [11], were initially characterized by their odors, which were considered to be more pleasant than other organic substances, considered as aliphatic species. Kekulé recognized that the common structural feature of these early aromatic compounds is a hexagonal ring of six carbon atoms [12]. The C_6_H_6_ formula of benzene, combined with the tetravalence of carbon, suggested a cyclohexatriene structure with six π-electrons in the three C=C double bonds. Thus, one of the four valence electrons from the carbon atom of each CH unit in benzene remains as a π electron after using two of the carbon valence electrons for bonding to adjacent carbon atoms and a third electron for bonding to the external hydrogen atom.

A noteworthy feature of benzene is the unusually low chemical reactivity of its C=C double bonds as compared, for example, with the cyclic tetraolefin cyclooctatetraene. Hückel [13] used molecular orbital theory to show that planar hydrocarbons with six π-electrons are delocalized systems exhibiting special stability, as indicated by a large HOMO-LUMO gap [14,15,16,17]. More generally, Hückel predicted special stability for planar cyclic hydrocarbons having 4*k* + 2 π-electrons, where *k* is an integer. The six π-electrons in benzene, as well as in the unusually stable ionic species tropylium (C_7_H_7_^+^) and cyclopentadienide (C_5_H_5_^−^), correspond to 4*k* + 2 where *k* = 1. The strength of the interaction between the π-orbitals on adjacent vertices linked by direct C–C bonds in such aromatic systems is characterized by a parameter *β*.

During the past several decades the concept of aromaticity has extended far beyond the original examples of planar cyclic hydrocarbons and their derivatives. Deltahedral boranes [18,19] and carboranes [20] of the type B*_n_*H*_n_*^2−^, CB*_n_*_–1_H*_n_*^−^, and C_2_B*_n_*_−2_H*_n_*, and their substitution products provide examples of three-dimensional aromatic systems [21]. A multicenter two-electron bond in the center of the deltahedra of such species corresponds to the π-bonding in planar cyclic hydrocarbons. Thus, the deltahedral boranes and carboranes fit into the 4*k* + 2 delocalized electron scheme where *k* = 0. The *β* values for the interaction between adjacent vertices in aromatic deltahedral boranes, which are linked by direct B–B, B–C, or C–C bonds, are similar to those in planar cyclic hydrocarbons.

Metal-metal interactions involving metal d orbitals that are related to aromaticity can occur in certain groups of polyoxometalates [22]. However, these interactions are much weaker since the metals involved are not directly bonded to each other in polyoxometalate structures. Thus, the metal polyhedra in polyoxometalates can be considered as macropolyhedra with relatively long edges consisting of M–O–M units rather than direct M–M bonds. This leads to considerably lower *β* values as compared with planar cyclic hydrocarbons or deltahedral boranes. As a result of the low *β* value, complete spin pairing of the delocalized electrons does not occur in polyoxometalates thereby leading to higher spin state analogues of aromatic systems. Thus the distinction between the aromaticity in diamagnetic planar cyclic hydrocarbons and deltahedral boranes and related delocalization in paramagnetic polyoxometalates is analogous to the distinction between low-spin coordination complexes with high ligand field splitting, such as Fe(CN)_6_^3−^ with one unpaired electron, and high-spin coordination complexes with low ligand field splitting, such as FeF_6_^3−^ with five unpaired electrons [23].

**Figure 1:**
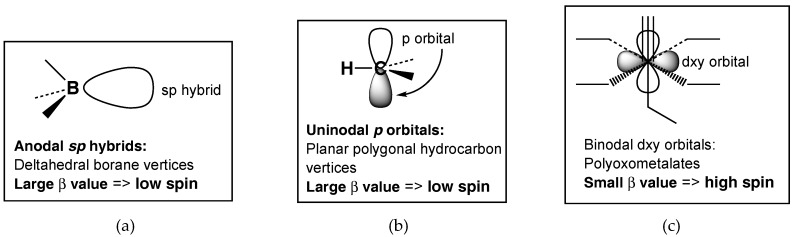
The relationship between the atomic orbitals involved in aromatic and other delocalized systems and the relative values of the interaction parameter *β*. (**a**) Deltahedral borane systems; (**b**) Planar polygonal hydrocarbon systems; (**c**) Polyoxometallate systems including vanadoborates.

Systems displaying aromaticity and related delocalization can be classified by the nodality of the interacting orbitals. The two-dimensional aromaticity in planar polygonal hydrocarbons involves interaction between carbon *p* orbitals with a single node, and thus can be considered as uninodal aromaticity (Figure 1). Similarly, the three-dimensional aromaticity in deltahedral boranes involves interaction between boron and carbon *sp* hybrid orbitals in the center of the deltahedron. Since these *sp* hybrid orbitals have no nodes, such aromaticity can be considered as anodal aromaticity. In both uninodal and anodal aromaticity, the interacting orbitals are located on adjacent directly bonded atoms leading to relatively large values of the interaction parameter *β*. This interaction parameter is larger than the electron repulsion energy, thereby leading to low-spin systems.

In the vanadoborates as in other polyoxometalates the interacting orbitals on each transition metal are *d* orbitals that have two nodes (Figure 1). However, the transition metals in such systems are not directly bonded to each other, but are separated by oxygen bridges. Therefore, the binodal interactions between transition metal atoms in polyoxometalates, including vanadium (IV) atoms in vanadoborates, are relatively weak interactions corresponding to low values of the interaction parameter *β*. Such low values of *β* are comparable to electron-electron repulsion energies, leading to high spin systems.

## 3. The High-Spin Vanadoborate Analogue of Benzene

Since the publication of my previous paper in 2001 [1] additional examples of vanadoborates have been synthesized. The known vanadoborates have now been classified into the following four categories [24]:

(1) V_6_B_20_ species containing a V_6_ macrohexagon imbedded in a B_20_ borate matrix, typically corresponding to a [V_6_B_20_O_50_H_8_]^8−^ anion building block [2,3,25].
(2) V_10_B_28_ species containing a V_10_ macrodecagon imbedded in a B_28_ borate matrix, typically corresponding to a [V_10_B_28_O_74_H_8_]^16−^ building block [5,6].
(3) V_12_B_18_ species containing a planar V_12_ unit imbedded in a B_18_ borate matrix, typically corresponding to a [V_12_B_18_O_60_]^6−^ building block [6,26].
(4) V_12_B_16_ species containing a three-dimensional V_12_ polyhedron imbedded in a B_16_ borate matrix, typically corresponding to a [V_12_B_16_O_50_(OH)_8_]^12−^ building block [27].

In the V_6_B_20_, V_10_B_28_, and V_12_B_16_ species all vanadium atoms are in the d^1^ +4 oxidation state and thus can provide an electron for delocalization throughout the vanadium cluster similar to the single electron provided CH vertices in benzene. However, in the V_12_B_18_ species, all of the vanadium atoms are in the d^0^ +5 oxidation state and thus do not have electrons available for delocalization. These +5 oxidation state vanadium atoms are analogous to the carbon atoms in the CH_2_ groups in a saturated cyclic hydrocarbon such as cyclohexane. Furthermore, the first two vanadoborate types, namely V_6_B_20_ and V_10_B_28_, can be regarded as high-spin analogues of planar cyclic hydrocarbons.

**Figure 2:**
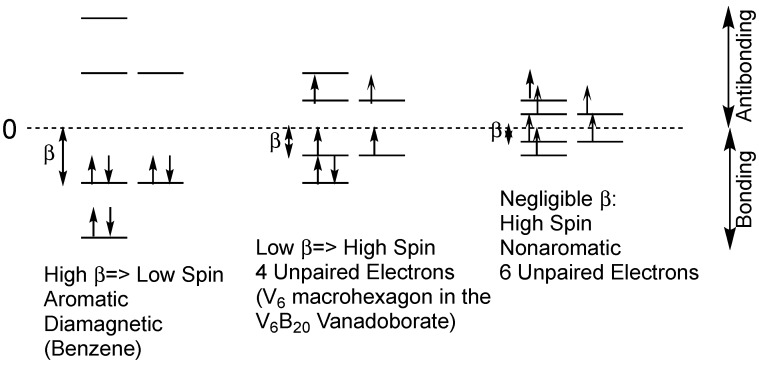
Comparison of the energy levels and spin states in benzene with those in the V_6_ macrohexagon in the V_6_B_20_ vanadoborates such as [V_6_B_20_O_50_H_8_]^8−^.

The relationship between the energy levels in benzene and those in the V_6_B_20_ vanadoborates is illustrated in Figure 2. In benzene the *β* value is large when compared with the spin pairing repulsion analogous to a strong ligand field in a low-spin coordination complex. All six π-electrons in benzene are paired leading to a diamagnetic species. However, in the V_6_B_20_ vanadoborates, the low *β* value is comparable to the spin pairing repulsion so that only partial spin pairing occurs, leading to a paramagnetic species. The experimental magnetic moment of [V_6_B_20_O_50_H_8_]^8−^ of 4.1 µ_B_ is close to the spin-only value of 4.9 µ_B_ for four unpaired electrons. Note that for a low *β* value that is small as compared with electron-electron repulsion energy can theoretically lead to a non-aromatic hexagonal system with six unpaired electrons (Figure 2).

## 4. The High-Spin Vanadoborate Analogue of [10]Annulene

**Figure 3:**
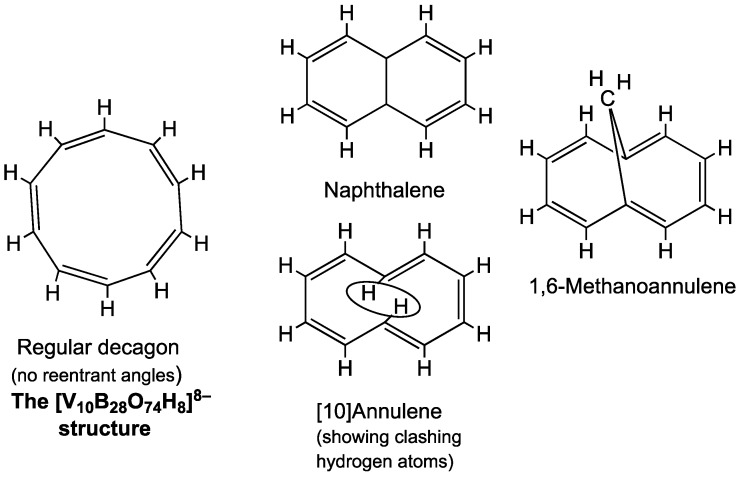
The structures of [10]annulene and related species.

The next member of the 4*k* + 2 π-electron series after benzene is cyclodecapentaene, also known more compactly as [10]annulene [9]. [10]Annulene has 10 π-electrons corresponding to 4*k* + 2 for *k* = 2. However, a regular C_10_H_10_ decagon with alternating C=C double bonds in a Kekulé-like structure for [10]annulene has unfavorable C–C–C angles, leading to considerable strain (Figure 3). Instead an arrangement of the ten carbons in the 10-membered C_10_ ring that is similar to that in naphthalene (C_10_H_8_) with some reentrant C–C–C angles has less angular strain. However, even that arrangement has a problem with transannular steric interference between the hydrogen atoms located at the pair of carbon atoms at the center of the two reentrant angles of the C_10_ ring. This difficulty can be overcome by replacing the offending hydrogen atoms with a transannular –CH_2_– bridge leading to the stable hydrocarbon 1,6-methano[10]annulene [9].

The V_10_B_28_ anion [V_10_B_28_O_74_H_8_]^16−^, found in species such as [Zn(OH_2_)(en)]_4_[Zn_4_(B_2_O_4_H_2_)(BO_2_H)_2_(V_10_B_28_O_74_H_8_)]·10H_2_O, is the vanadoborate analogue of [10]annulene. However, the central V_10_ system in this structure is essentially a regular decagon rather than a 10-membered naphthalene-like ring with reentrant angles similar to 1,6-methano[10]annulene. Thus the borate matrix combined with the flexibility of the V–O–V edges in the V_10_ ring keeps the V_10_ ring in a macrodecagon configuration without any reentrant angles.

**Figure 4:**
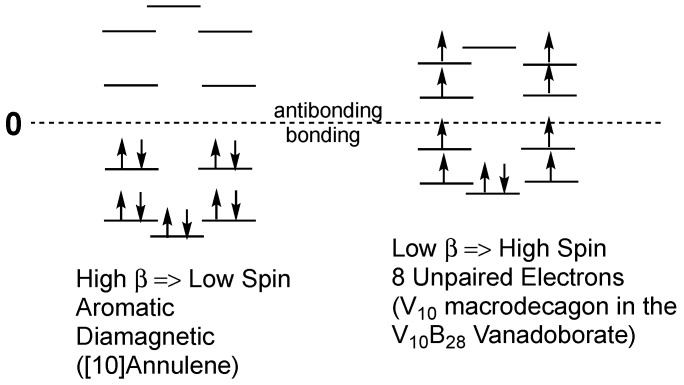
Comparison of the energy levels and spin states in [10]annulene and in the V_10_ macrodecagon in the V_10_B_28_ vanadoborates, such as [V_10_B_28_O_74_H_8_]^16−^.

The electronic properties of the V_10_ ring in the [V_10_B_28_O_74_H_8_]^16–^ anion appear to be analogous to those of the V_6_ ring in the [V_6_B_20_O_50_H_8_]^8−^ anion discussed above, but with an additional layer of doubly degenerate orbitals in the molecular orbital pattern of the delocalization in the V_10_ system (Figure 4). The experimental magnetic moment of 0.961 µ_B_ per vanadium atom corresponds to ~9.6 µ_B_ for the entire V_10_ system. This suggests a low *β* value corresponding approximately to eight unpaired electrons with an electron pair in only the lowest energy non-degenerate orbital. However, the decrease of the χ_M_*T* value to nearly zero obtained upon cooling to 2 K suggests that this system becomes diamagnetic with complete electron pairing at low temperatures.

Comparison of Figure 2 and Figure 4 show a common feature of the patterns of the macropolygon molecular orbitals electron distributions in the V_6_ macrohexagons in [V_6_B_20_O_50_H_8_]^8−^ and the V_10_ macrodecagons in [V_10_B_28_O_74_H_8_]^16−^. These patterns relate to the experimentally observed magnetic properties of these systems. Thus, for both V_6_B_20_ and V_10_B_28_, the non-degenerate lowest energy molecular orbital still retains an electron pair whereas all of the higher lying doubly degenerate orbitals are half-filled with single electrons.

## 5. Conclusions

The vanadium-vanadium interactions in the polygonal aggregates of d^1^ vanadium(IV) atoms, with a total of 4*k* + 2 vanadium electrons (*k* an integer) imbedded in an electronically inactive borate matrix in certain vanadoborate structures are analogous to the ring carbon-carbon interactions in diamagnetic planar cyclic hydrocarbons. They can be interpreted as representing a high-spin analogue of aromaticity. Thus, the vanadoborate anion [V_6_B_20_O_50_H_8_]^8−^ with six V(IV) electrons (i.e., 4*k* + 2 for *k* = 1) contains a macrohexagon of d^1^ V(IV) atoms with only two of the six V(IV) electrons being paired, leading to four unpaired electrons. This is related to the experimental magnetic moment of [V_6_B_20_O_50_H_8_]^8−^ of 4.1 µ_B_. Similarly, the vanadoborate anion [V_10_B_28_O_74_H_8_]^16−^ with ten V(IV) electrons (i.e., 4*k* + 2 for *k* = 2) contains a macrodecagon of d^1^ V(IV) atoms with eight unpaired electrons. Again, this high-spin system is related to the aromaticity in the diamagnetic 1,6-methano[10]annulene having ten π electrons. This is related to the experimental total magnetic moment of ~9.6 µ_B_ for the 10 vanadium atoms in [V_10_B_28_O_74_H_8_]^16−^.
